# Treatment of Diphtheria

**Published:** 1896-03

**Authors:** Frederic W. Bartlett

**Affiliations:** Buffalo, N. Y.


### PRESIDENT'S ANNUAL ADDRESS.[1]

### TREATMENT OF DIPHTHERIA.

By FREDERIC W. BARTLETT, M. D., Buffalo, N. Y.

UNDER the shadow of a great physical affliction I am compelled to forego what would have been to me one of the most pleasant acts of my life, the delivery of the annual presidential address, and to avail myself of the kind offer of the secretary to read this paper,—one of the many evidences of his good-will during our official connection.

Forty years ago I took up my residence in Buffalo, and within that period almost all of the great evolutions of our art have occurred; not only with ourselves as a profession, but in all that exalts and embellishes civilised life, the most wonderful progress has been made.

Leaving to various societies the task which remained to them, and referring scarcely at all to the more popular phrase, "familiar to our ears as household words," my thoughts turn to the results of special scientific investigation and experiments dealing with the very origin and methods of life hidden through all the ages, to the days succeeding Jenner, whose almost accidental observation not only relieved the world of the continual ravages of an awful pestilence, but ultimately introduced through the scientific studies and experiments of Pasteur and other notable coadjutors and successors, after years of comparative slumber, hypodermic antitoxins, whose value and importance are now beginning to be made known.

In this paper I report only a few cases of diphtheria. Twenty-seven years ago I was called to see a child sick with diphtheria, on Elk street, another child then lying dead in the house from the same malady. The disease had invaded the larynx. Across the street

1. Read at the annual meeting of the Medical Society of the County of Erie, January 14, 1896.

was a tin shop, where I had a small tin tube made, ten inches in length, one-quarter inch in diameter, with a slight curve at one end. With this I introduced into the fauces finely powdered table salt, a small quantity, equivalent to an ordinary hypodermic tablet, at the same time raising a small blister over the nuchæ, about the size of half a dollar. This was dressed every three or four hours with lard, to which a few grains of fine salt had been added. By this application a free discharge was created, the laryngitis was relieved and the patient made a good recovery.

I subsequently reported this case, with nine others, to the Buffalo Medical and Surgical Association,—eight being cures, two deaths from relapses, respectively, at the end of one and two weeks. Since that date I have reported modifications of the throat application. Later I used powdered quinine, tannin and sulphur, and later still I used a powder of which I give the formula:

| | | |
|---|---|---|
| Quinine | gr. | xx. |
| Menthol | " | x. |
| Thymol | " | x. |
| Pulv. sulphur | " | xx. |
| Pulv. marsh-mallow or starch | ʒ | ii. |

This powder, when used, should be rubbed up each time, as it granulates coarsely, the menthol and thymol being apt to make it sticky.

I question whether caustics, either in solution or solids, are of any real use as an application in diphtheria. What we want is a soluble germicide applied in powder or spray form.

Within the past year I have treated six cases with the application given above, with prompt success, so prompt indeed that the variety of the disease might be doubted had it not been confirmed by the bacteriologist of the health department. The effect of the application was uniform in every case. These applications were made every fourth hour. After the first one, there is generally an arrest of the spread of the exudate, while the third or fourth rolls up in appearance like light wool and is expelled, leaving only the clean ulcer or ulcers to be treated with the general application somewhat longer.

The *modus operandi* is as follows: take a leaf from a letter pad of good paper, a glazed surface being preferred, and use an ordinary wooden pencil, first removing the rubber to give it uniformity. Use the pencil to shape a tube from the paper; use the entire sheet if it is not very large, have an assistant wind thread around each end and also in the center, to prevent unrolling, then blow the pencil out or push it out with some smaller rod ; use of the powder deposited in the hollow of the left hand almost as much as would make a hypodermic tablet or small pea ; gather this into the end of the tube. An attendant securely holding the child, with a teaspoon or dessertspoon depress the tongue, then blow vigorously the powder into the fauces. Repeat every third or fourth hour, carefully using the end of the tube marked for the operator. If there are other children in the family, prepare separate tubes for each. Use in the same way on the preventive theory.

The preventive theory is this : diphtheria makes its incursion on a limited area, including one or both tonsils and the posterior fauces. This may indicate a simple impinging upon the surface in the act of inspiration, or something more—a *natural selection* for nutrition and reproduction. The powder used is purely empirical, but it is intended to illustrate experimentally the possibility of clothing the mucous surface with a germicide adhesive application inimical to *natural selection.* Assuming by repeated tests that the caustic germicides are too destructive in their local effects and too brief in the time allotted for a safe application to accomplish the death of the bacillus, we may endeavor by the continuous effect of application inhibitory to the reception, propagation, locomotion and transmission to form colonies or toxides in the circulation, we diminish the invading host and lessen the task suddenly imposed on the ordinary antitoxin provided by nature. The most important action of the artificial antitoxin introduced hypodermically changes wholly the prognosis ; the mucous membrane of the tonsils and fauces is no longer a field for the reception or propagation of the receptive germ, and since the area thus defined is the only one that has a natural selection by the bacillus, and all observations note this, it follows that promptness must soon very largely reduce the ravages of diphtheria. You will note the importance I attach to the changes in the propagation area, also the artificial antitoxin in the circulation. It is from this theory, the increased circulation in the skin, that I based my treatment of scarlet fever ; the dry mustard frictions increased activity of the circulation and the subsequent bichlorate bath returns the blood, minus a large proportion of its toxin, to pass through the capillaries and glands, inhibiting the activity of the disease.

In closing this paper, I thank the society, in retiring from office, for having conferred upon me the highest honor it is in its power to bestow.

